# Digital Health Technologies to Improve Medication Adherence and Treatment Outcomes in Patients With Tuberculosis: Systematic Review of Randomized Controlled Trials

**DOI:** 10.2196/33062

**Published:** 2022-02-23

**Authors:** Abdurahman Ridho, Sofa D Alfian, Job F M van Boven, Jutti Levita, Esin Aki Yalcin, Ly Le, Jan-Willem Alffenaar, Eelko Hak, Rizky Abdulah, Ivan S Pradipta

**Affiliations:** 1 Department of Pharmacology and Clinical Pharmacy Faculty of Pharmacy Universitas Padjadjaran Sumedang Indonesia; 2 Doctor Program in Pharmacy Faculty of Pharmacy Universitas Padjadjaran Sumedang Indonesia; 3 Center of Excellence in Higher Education for Pharmaceutical Care Innovation Universitas Padjadjaran Sumedang Indonesia; 4 Department of Clinical Pharmacy and Pharmacology University Medical Center Groningen University of Groningen Groningen Netherlands; 5 Medication Adherence Expertise Center of the Northern Netherlands Groningen Netherlands; 6 Department of Pharmaceutical Chemistry Faculty of Pharmacy Ankara University Ankara Turkey; 7 Vingroup Big Data Institute Hanoi Vietnam; 8 Faculty of Medicine and Health School of Pharmacy University of Sydney Sydney Australia; 9 Sydney Institute for Infectious Diseases Sydney Australia; 10 Westmead Hospital Sydney Australia; 11 Pharmacotherapy, Pharmacoepidemiology and Pharmacoeconomics Research Institute of Pharmacy University of Groningen Groningen Netherlands

**Keywords:** tuberculosis, intervention, eHealth, medication adherence technology, nonadherence, digital health, systematic review, treatment outcomes

## Abstract

**Background:**

Nonadherence to medication in tuberculosis (TB) hampers optimal treatment outcomes. Digital health technology (DHT) seems to be a promising approach to managing problems of nonadherence to medication and improving treatment outcomes.

**Objective:**

This paper systematically reviews the effect of DHT in improving medication adherence and treatment outcomes in patients with TB.

**Methods:**

A literature search in PubMed and Cochrane databases was conducted. Randomized controlled trials (RCTs) that analyzed the effect of DHT interventions on medication adherence outcomes (treatment completion, treatment adherence, missed doses, and noncompleted rate) and treatment outcomes (cure rate and smear conversion) were included. Adult patients with either active or latent TB infection were included. The Jadad score was used for evaluating the study quality. The PRISMA (Preferred Reporting Items for Systematic Reviews and Meta-Analyses) guideline was followed to report study findings.

**Results:**

In all, 16 RCTs were selected from 552 studies found, and 6 types of DHT interventions for TB were identified: 3 RCTs examined video directly observed therapy (VDOT), 1 examined video-observed therapy (VOT), 1 examined an ingestible sensor, 1 examined phone call reminders, 2 examined medication monitor boxes, and 8 examined SMS text message reminders. The outcomes used were treatment adherence, including treatment completion, treatment adherence, missed dose, and noncompleted rate, as well as clinical outcomes, including cure rate and smear conversion. In treatment completion, 4 RCTs (VDOT, VOT, ingestible sensor, SMS reminder) found significant effects, with odds ratios and relative risks (RRs) ranging from 1.10 to 7.69. Treatment adherence was increased in 1 study by SMS reminders (RR 1.05; 95% CI 1.04-1.06), and missed dose was reduced in 1 study by a medication monitor box (mean ratio 0.58; 95% CI 0.42-0.79). In contrast, 3 RCTs of VDOT and 3 RCTs of SMS reminders did not find significant effects for treatment completion. Moreover, no improvement was found in treatment adherence in 1 RCT of VDOT, missed dose in 1 RCT of SMS reminder, and noncompleted rate in 1 RCT of a monitor box, and 2 RCTs of SMS reminders. For clinical outcomes such as cure rate, 2 RCTs reported that phone calls (RR 1.30; 95% CI 1.07-1.59) and SMS reminders (OR 2.47; 95% CI 1.13-5.43) significantly affected cure rates. However, 3 RCTs found that SMS reminders did not have a significant impact on cure rate or smear conversion.

**Conclusions:**

It was found that DHT interventions can be a promising approach. However, the interventions exhibited variable effects regarding effect direction and the extent of improving TB medication adherence and clinical outcomes. Developing DHT interventions with personalized feedback is required to have a consistent and beneficial effect on medication adherence and outcomes among patients with TB.

## Introduction

Tuberculosis (TB), an infectious disease caused by *Mycobacterium tuberculosis* (M. tb), is one of the top 10 most deadly infectious diseases worldwide [1]. The M. tb pathogen can easily spread through air transmission by coughing or sneezing [1]. In 2019, it was estimated that 10 million people globally were infected with TB [1]. Therefore, long-term antibiotic treatment is needed to control TB infection and avoid disease spread.

Treatment for active drug-susceptible TB usually takes at least 6 months, while latent tuberculosis infection (LTBI) can be between 1 and 6 months [2]. The duration of treatment can be longer if the pathogen is resistant to either the first- or second-line of anti-TB medication [3]. Although effective TB medication is available, treatment is prone to nonadherence, resulting in treatment failure [2]. Numerous factors affect medication adherence, such as poor communication between patient and health care provider, socioeconomic status, health care system factors, patients’ mental condition, therapy features, and other patient factors [4,5]. Moreover, a high risk of nonadherence to the medication has been reported in patients with LTBI because they do not feel any signs or symptoms of the disease but do experience the side effects of the medication[2].

Previous studies have indicated that nonadherence to TB medication impacts clinical and economic TB outcomes [6]. Notably, the consequences of nonadherence include worsening of the disease [6,7] but also the development and spread of drug-resistant TB [7]. Although poor medication adherence is a widely significant problem in TB treatment, it cannot be managed easily due to the heterogeneity of the underlying factors [5].

Different digital health technologies (DHT) help manage patients with TB by monitoring and supporting their medication adherence [8]. Among them, SMS text message reminders and video directly observed therapy (VDOT) stand out [8]. On the one hand, past evidence indicates that their effect concerning supporting medication adherence among patients with TB is questionable [9,10]. On the other hand, the field of DHTs is evolving rapidly, and some more recent developments may provide more promising approaches in the management of medication adherence. An updated, comprehensive analysis is needed to analyze the effect and potential development of DHTs for managing medication adherence in patients with TB. Therefore, this study systematically reviewed the effect and potential development of various DHTs in improving medication adherence and treatment outcomes in patients with TB.

## Methods

### Study Design

Randomized controlled studies (RCTs) indexed in the PubMed and Cochrane databases were systematically reviewed following the PRISMA (Preferred Reporting Items for Systematic Reviews and Meta-Analysis) guidelines [11,12] (Multimedia Appendix 1).

### Data Sources

As a reputable databases in the medical field, PubMed was selected to effectively obtain qualified RCTs. Additionally, the Cochrane Database was included since it provides published and unpublished interventional studies.

### Inclusion and Exclusion Criteria

RCTs published in English between March 2002 and January 2020 focusing on interventions using DHT for improving treatment outcomes and medication adherence in patients with TB were included in this review. The time restriction was defined using the leading World Health Organization (WHO) report from 2003 on interventional strategies for improving medication adherence as the starting point [4]. Studies that did not measure the effect of the intervention, had no comparison, applied a different study design, were not original studies (eg, study protocol, review), or were reported as an abstract only were excluded. The population, intervention, comparison, and outcome were predefined to collect articles according to the study's objective.

### Population

The study population comprised patients aged 15 years or older with all types of TB (active TB, LTBI, pulmonary TB, extrapulmonary TB, drug-sensitive TB, or drug-resistant TB). TB status needed to be confirmed using laboratory or clinical testing, such as Mantoux, TB symptoms, chest radiography, interferon gamma release assays, and smear or sputum test; or other examinations, such as polymerase chain reaction or phenotypic drug susceptibility testing.

### Interventions and Comparisons

We included studies evaluating the effect of DHTs, such as smartphone apps, video observation, phone reminders, ingestible sensors, SMS reminders, and other digital health interventions, that aimed to improve medication adherence and TB treatment outcomes. All selected studies had a comparison group with patients receiving usual care (mainly traditional directly observed therapy [DOT]) to measure the incremental effect of the intervention.

### Outcome Measures

The primary outcome was medication adherence (ie, treatment completion, adherence rate, and missed doses), while the secondary outcome included clinical outcomes (ie, cure rate and smear sputum conversion rate).

Medication adherence could be measured using self-report on the device directly (VDOT) and indirectly (VOT), responding to phone calls and SMS text messages, detecting drug taking through a monitor box, or pill counting. Notably, medication adherence to (respiratory) medicines consists of 3 phases: initiation, implementation, and persistence [13] according to the global TB definition [14] and the ESPACOMP Medication Adherence Reporting Guideline (EMERGE) [15]. In this study, the adherence rate was defined as the rate of anti-TB medication taken by a patient with TB with treatment completion. Following the WHO definition, completion treatment was defined as a patient with TB who completed treatment without evidence of failure (no record of sputum smear or culture test results in the last month of treatment). Hence, the adherence rate was operationally defined as the implementation phase in this study. Moreover, the persistence phase was defined as completion treatment, and noncompletion treatment was deemed to be similar to nonpersistence. Noncompletion treatment was defined as a patient with TB who discontinued the medication before the last defined dose [16,17]. Missed doses were included in the implementation phase and were defined as the percentage of monthly TB medicines missed as measured according to pill count and failure to open the medication box, where the minimum percentage of missed doses was 20% [18].

The clinical outcomes included cure rate, with cure being defined as M. tb culture–positive results at the beginning and negative results in the last month of treatment and on at least 1 previous occasion. The other clinical outcome was sputum conversion, which was defined as the conversion of positive to negative M. tb culture during the treatment [14].

### Search Strategies

Specific key terms related to the study population, intervention, comparison, outcome, and design were developed for the specific databases used. The search strategies for the PubMed and Cochrane databases are described in the following sections.

#### PubMed Database

The search strategy for PubMed was as follows: (“technology”[tw] OR “digital adherence”[tw] OR “mHealth”[tw] OR “mobile health”[tw] OR “mobile app”[tw] OR “mobile apps”[tw] OR “mobile application”[tw]) AND (“medication adherence” [Mesh] OR “adherence”[tw] OR “concordance”[tw] OR “compliance”[tw] OR “nonadherence”[tw] OR “noncompliance”[tw] OR “nonconcordance”[tw]) AND (“tuberculosis/drug therapy”[Mesh] OR “tuberculosis infection”[tw] OR “tb”[tw] OR “active tuberculosis”[tw] OR “latent tuberculosis”[tw] OR “pulmonary tuberculosis”[tw] OR “extrapulmonary tuberculosis”[tw]).

#### Cochrane Database

The search strategy for Cochrane Database was as follows: (“technology” OR “digital adherence” OR “mHealth” OR “mobile health” OR “mobile app” OR “mobile apps” OR “mobile application”) AND (“medication adherence” [Mesh] OR “adherence” OR “concordance” OR “compliance” OR “nonadherence” OR “noncompliance” OR “nonconcordance”) AND (“tuberculosis/drug therapy” [Mesh] OR “tuberculosis infection” OR “tb” OR “active tuberculosis” OR “latent tuberculosis” OR “pulmonary tuberculosis” OR “extrapulmonary tuberculosis”).

### Data Selection, Collection, and Extraction

AR conducted eligibility evaluation based on the title and abstract. The full texts of potentially eligible articles were retrieved and assessed by AR. ISP and SDA conducted further independent verification of the abstract and full-text screening. Any disagreements among the reviewers (AR, ISP, and SDA) were resolved by discussion. Data from the selected articles were extracted by AR and then verified by ISP for relevant information, such as publication year, type of DHT intervention, setting, population, study outcome, and comparison groups.

### Summary Measures and Synthesis of Results

In the case of comparable homogenous studies being retrieved, quantitative data synthesis was considered. However, a qualitative narrative review was used in case the data were heterogenous in terms of the population, intervention, comparisons, or outcomes. Several point estimates were considered for the data analysis, such as the mean ratio for continuous outcome data and the relative risk (RR) and odds ratio (OR) for dichotomous outcome data with a 95% CI.

### Quality Assessment of the Included Articles

As all included articles were RCTs, the Jadad score was used to assess the individual articles [19]. The Jadad score has 3 assessment domains: randomization, blinding method, and participant withdrawal with a minimum score of 1 (poor quality) and a maximum score of 5 (good quality).

## Results

### Study Selection

**Figure 1:**
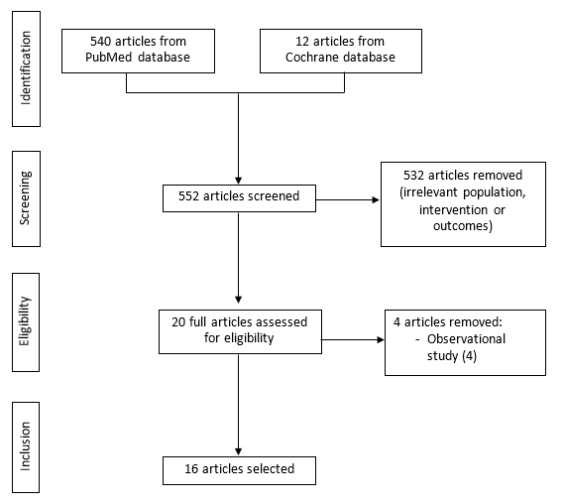
Flow diagram of the article selection process.

Our literature search initially identified 552 articles. After removal of duplicates, the screening of titles and abstracts yielded 20 relevant articles. The full-text screening process resulted in a final study sample of 16 articles. Variations in the study population, comparisons, and outcomes across the included studies were identified; therefore, quantitative analysis could not be conducted in this study considering the heterogeneity. The flow diagram and the screening process are provided in Figure 1.

The 16 included studies were conducted in different countries: England [20], USA [21-23], Taiwan [24], China [17,25], Pakistan [26,27], South Africa [9], Cameroon [28], Canada [10], Argentina [29], Sudan [17], Thailand [30], and Haiti [31].

The median sample size of the source population was 259 participants, ranging from 37 participants [29] to 1110 participants [24]. Most studies targeted either patients alone (n=12) or patients and health care professionals (n=4). Several study designs were used in the included articles: 11 RCTs with a 2-arm design [10,17,21-24,26,27,29-31], 4 with a 2-arm cluster design [9,20,25,28], and 1 with a 4-arm cluster design [17].

Across the studies, multiple various interventions were assessed, including 3 assessing VDOT [21,22,24], 1 VOT [20], 1 phone call reminders [30], 2 medication monitor boxes [18,31], 1 ingestible sensors [23], and 8 SMS reminders [9,10,17,25-29]. The characteristics of all included articles are indicated in Table 1.

**Table 1:** Characteristics of the included articles.

No.	Authors, publication year	Target	Study period	Setting	Intervention	Participants in the intervention and comparison group, n	Study outcomes	Outcome measurements
1	Story et al, 2019 [20]	Patients	Sept 2014 to Oct 2016	22 clinics in England (UK)	VOT^a^ intervention in which patients record and send videos of every dose ingested and adverse medicine events using the smart app^b^	112 and 114	Treatment completion	Scheduled treatment observation
2	Chuck et al, 2016 [21]	Patients	Sept 2013 to Sept 2014	TB^c^ clinics in New York City, USA	Patients swallowing a pill in front of a camera on their schedule on VDOT^d^ calls^b^	49 and 267	Treatment completion	Scheduled VDOT^d^ session
3	Lam et al, 2018 [22]	Patients	Feb to Oct 2015	4 New York City health department TB clinics, New York City, USA	Patients taking medicine observed by a VDOT worker^b^	50 and 302	Treatment completion	Scheduled VDOT session
4	Chen et al, 2020 [24]	Patients and HCP^e^	Jan 2014 to Dec 2017	Health facilities in Taipei, Taiwan	Patients taking medicine under 2-way video calls^b^	80 and 160	Treatment adherence	Scheduled VDOT session
5	Kunawararak et al, 2011 [30]	Patients	Apr 2008 to Dec 2009	Public hospitals in 7 provinces of Northern Thailand	Patients receiving phone call reminders to take their medication^b^	30 and 30	Cure rate	Negative culture result at the end of treatment
6	Liu et al, 2015 [18]	Patients	June 2011 to Mar 2012	36 districts within the province of Heilongjiang, Jiangsu, Hunan, and Chongqing, China	Patients receiving a reminder via SMS, medication monitor box, or their medication^b^	3069 and 1104	Missed doses	Pill count and failure to open the medication monitor box
7	Moulding & Caymittes, 2002 [31]	Patients	July 1983 to Nov 1985	Clinic at Port-Au-Prince, Haiti	Medication monitor and counseling in which the medication monitor is based on moving a minute piece of uranium along a strip of photographic film to record the interval between the removal of each tablet of medication^f^	64 and 127	Noncompleted rate	Counting the number of dots on the film strip
8	Browne et al, 2019 [23]	Patients	Oct 2013 to Jan2017	San Diego and Orange County divisions of TB Control and Refugee Health, USA	Patients given the WOT^g^ IS-Rifamate (ingestible sensor)^i^	41 and 20	Treatment completion	The number of doses confirmed
9	Fang et al, 2017 [25]	Patients	Dec 2014 to Dec 2015	6 districts from Anhui province, China	An SMS reminder sent once per day to remind patients for taking the medicine, reexamining the physical condition, and improving knowledge^b^	160 and 190	Treatment completion and missed doses	Pill counting
10	Mohammed et al, 2016 [26]	Patients and HCP^e^	Mar 2011 to Feb 2014	Public and private sector tuberculosis clinics in Karachi, Pakistan	Daily SMS reminders sent to participants who are asked to respond through SMS or missed calls after taking their medication^b^	1110 and 1097	Treatment completion and smear conversion	Pill counting and smear conversion at the end of treatment
11	Belknap et al, 2017 [9]	Patients	Sept 2012 to Apr 2014	Outpatient tuberculosis clinics in the USA, Spain, Hong Kong, and South Africa	SAT^h^ used without reminder and SAT used with weekly SMS reminder^b^	315 and 328	Treatment completion	Pill counting and self-report
12	Bediang et al, 2018 [28]	Patients	Feb 2013 to Apr 2014	Treatment and Diagnostic Centres of Yaoundé, Cameroon	Daily SMS reminder for 6 months^b^	137 and 142	Treatment completion and cure rate	Pill counting and negative culture result at the end of treatment
13	Johnston et al, 2018 [10]	Patients and HCP	June 2012 to Sept 2015	TB clinics in British Columbia, Canada	2-way weekly SMS reminder to patients who need to respond within 48 hours^i^	170 and 188	Treatment completion	Pill counting and self-report
14	Iribarren et al, 2013 [29]	Patients and HCP	Nov 2011 to June 2012	Clinic located within Health Region V in the province of Buenos Aires, Argentina	Patients receiving SMS reminders every day and being required to text after medication administration^i^	18 and 19	Treatment completion and treatment adherence	Pill counting and self-report
15	Ali & Martin, 2019 [17]	Patients	May 2017 to Mar 2018	8 TB treatment units in Khartoum Province, Khartoum State, Sudan	Patients receiving standard of care, with the additional text messages every 48 hours during the first 2 months and weekly thereafter until the end of treatment^i^	74 and 74	Cured rate and noncompleted rate	Negative culture result at the end of treatment and pill counting
16	Farooqi et al, 2017 [27]	Patients	June 2014 to June 2015	TB clinics of Khyber Teaching Hospital Peshawar and Emergency Satellite Hospital Nahaqi, Pakistan	Patients receiving daily SMS reminders and DOT^b^	74 and 74	Cure rate and noncompleted rate	Negative culture result at the end of treatment and pill counting

^a^VOT: video-observed treatment.

^b^The comparison group is the directly observed treatment (DOT).

^c^TB: tuberculosis.

^d^VDOT: video directly observed treatment.

^e^HCP: health care provider.

^f^The comparison group is the simple container.

^g^WOT: wirelessly observed therapy.

^h^SAT: self-administered therapy.

^i^The comparison group is the standard of care.

### Outcomes

The medication adherence-related outcomes reported across the 16 RCTs were treatment completion in 10 (63%) studies [9,10,20-23,25,26,28,29], treatment adherence in 2 (13%) [24,29], missed doses in 2 (13%) [18,25], and noncompleted rate in 3 (19%) [17,27,31]. Note that some RCTs reported more than 1 adherence outcome. Clinical outcomes included cure rate in 4 RCTs (25%) [17,27,28,30] and smear conversion in 1 RCT [26] (6%).

#### Adherence Outcomes: Positive Findings

Four RCTs showed that treatment completion was significantly and positively impacted by a DHT intervention, with effect sizes varying by type of intervention. Notably, VDOT or VOT was found to be more effective compared to DOT in 2 studies from the UK and USA (UK: OR 2.52; 95% CI 1.17-5.54 [20]; USA: RR 1.36; 95% CI 1.19-1.55 [22]). Furthermore, studies on ingestible sensors (OR 7.69; 95% CI 4.51-14.48) [23] and 1 of the SMS reminder intervention studies (RR 1.1; 95% CI 1.04–1.18) [25] reported positive effects on treatment completion.

The outcome of treatment adherence was increased in 1 study that evaluated SMS reminders (RR 1.05; 95% CI 1.04-1.06) [29], and missed doses was reduced in 1 study that used a medication monitor box (mean ratio 0.58; 95% CI 0.42-0.79) [18] in patients with TB.

#### Adherence Outcomes: Negative Findings

Beyond the trials that reported the positive impact of DHT interventions on adherence outcomes, several studies that did not report any significant effect on medication adherence outcomes were also identified. Three RCTs did not find any significant effect on treatment completion from VDOT (RR 0.99, 95% CI 0.93-1.05 [21]; RR 1.00, 95% CI 0.79-1.26 [26]; RR 0.87, 95% CI 0.81-0.94 [9]), and another 3 indicated a lack of evidence for a positive effect of SMS reminders (OR 1.45, 95% CI 0.81-2.56 [28]; RR 0.97, 95% CI 0.88-1.07 [10]; RR 0.99, 95% CI 0.93-1.05 [29]).

Additionally, treatment adherence was not impacted by VDOT in 1 RCT (RR 1.08; 95% CI 0.89-1.32 [24]), and neither were missed doses by SMS reminders (RR 0.40; 95% CI 0.11-1.50 [25]), or the noncompleted rate by a monitor box (RR 0.55; 95% CI 0.21-1.42 [31]), or SMS reminders (OR 1.67, 95% CI 0.52-5.37 [17]; RR 0.76, 95% CI 0.18-3.28 [27]).

#### Clinical Outcomes: Positive Findings

In 2 studies, the clinical outcome of cure rate was positively affected by DHT [17,30]. It was observed that cure rate was increased through SMS reminders (OR 2.47, 95% CI 1.13-5.43 [17]) and phone call reminders (RR 1.30, 95% CI 1.07-1.59 [30]) in patients with TB.

#### Clinical Outcomes: Negative Findings

Three studies did not find there to be a significant effect of DHT on clinical outcomes, with 2 RCTs indicating no effects of SMS reminders on cure rate (OR 1.06;,95% CI 0.65-1.73 [28]; RR 1.05, 95% CI 0.62-1.76 [27]) and 1 study indicating no effect of SMS reminders on smear conversion (RR 1.00, 95% CI 0.90-1.12) [26]. The effects of interventions are summarized in Table 2.

**Table 2:** Effects of digital health technology interventions on medication adherence and clinical tuberculosis outcomes.

No.	First author, year of publication	Intervention^a^	Medication adherence outcome (95% CI)				Clinical outcome (95% CI)		
			Treatment completion	Treatment adherence	Missed doses	Noncompleted rate	Cure rate	Smear sputum conversion	
1	Story et al, 2019 [20]	VOT^b^	OR^c^: 2.52 (1.17-5.54)	N/A^d^	N/A	N/A	N/A	N/A	
2	Chuck et al, 2016 [21]	VDOT^e^	RR^f^: 0.99 (0.93-1.05)	N/A	N/A	N/A	N/A	N/A	
3	Lam et al, 2018 [22]	VDOT	RR: 1.36 (1.19-1.55)	N/A	N/A	N/A	N/A	N/A	
4	Chen et al, 2020 [24]	VDOT	N/A	RR: 1.08 (0.89-1.32)	N/A	N/A	N/A	N/A	
5	Kunawararak et a., 2011 [30]	Phone call reminder	N/A	N/A	N/A	N/A	RR: 1.30 (1.07-1.59)	N/A	
6	Liu et al, 2015 [18]	SMS, medication monitor box, and combined	N/A	N/A	MR^g^: 0.94 (0.71-1.24) MR: 0.58 (0.42-0.79) MR: 0.49 (0.27-0.88)	N/A	N/A	N/A	
7	Moulding & Caymittes, 2002 [31]	Medication monitor with counseling	N/A	N/A	N/A	RR: 0.55 (0.21-1.42)	N/A	N/A	
8	Browne et al, 2019 [23]	Ingestible sensor	OR: 7.69 (4.51-14.48)	N/A	N/A	N/A	N/A	N/A	
9	Fang et al, 2017 [25]	SMS reminder	RR: 1.10 (1.04-1.18)	N/A	RR: 0.40 (0.11-1.50)	N/A	N/A	N/A	
10	Mohammed et al, 2016 [26]	SMS reminder	RR: 1.00 (0.79–1.26)	N/A	N/A	N/A	N/A	RR: 1.00 (0.90–1.12)	
11	Belknap et al, 2017 [9]	SMS reminder	RR: 0.87 (0.81-0.94)	N/A	N/A	N/A	N/A	N/A	
12	Bediang et al., 2018 [28]	SMS reminder	OR: 1.45 (0.81-2.56)	N/A	N/A	N/A	OR: 1.06 (0.65-1.73)	N/A	
13	Johnston et al, 2018 [10]	SMS reminder	RR: 0.97 (0.88-1.07)	N/A	N/A	N/A	N/A	N/A	
14	Iribarren et al, 2013 [29]	SMS reminder	RR: 0.99 (0.93-1.05)	RR: 1.05 (1.04-1.06)	N/A	N/A	N/A	N/A	
15	Ali & Martin, 2019 [17]	SMS reminder	N/A	N/A	N/A	OR 1.67 (0.52-5.37)	OR: 2.47 (1.13-5.43)	N/A	
16	Farooqi et al, 2017 [27]	SMS reminder	N/A	N/A	N/A	RR: 0.76 (0.18-3.28)	RR: 1.05 (0.62-1.76)	N/A	

^a^The comparison group is provided in Table 1.

^b^VOT: video-observed therapy.

^c^OR: odds ratio.

^d^N/A: not applicable.

^e^VDOT: video directly observed therapy.

^f^RR: relative risk.

^g^MR: mean ratio.

### Quality Assessment of the Included Articles

None of the included studies had the maximum Jadad score (5 points). Eleven studies (69%) had a relatively high score (3 points) since they appropriately described the randomization method and clearly illustrated the withdrawals of participants [9,10,18,20,23,25-29,31]. Furthermore, 5 of the 16 studies (31%) had lower Jadad scores (2 points) due to the absence of a description of the randomization procedure [17,21,22,24,30]. The risk of bias assessment is indicated in (Multimedia Appendix 2).

### Data Synthesis

As shown in Table 1, considerable variations in the study population, comparisons, and outcomes across the included studies were identified. Therefore, the quantitative analysis could not be conducted considering the heterogeneity observed.

## Discussion

### Main Findings

This systematic review indicated that DHT interventions, including VOT, VDOT, medication monitor boxes, ingestible sensors, and SMS reminders, could effectively improve medication adherence in patients with TB. However, half of the 4 V(D)OT and over half of the 8 SMS reminder studies showed no significant effect on adherence outcomes, resulting in inconclusive evidence regarding their effectiveness with respect to the extent of improving medication adherence. Regarding reported clinical outcomes, 1 study with phone call reminders and 1 of the 3 studies with SMS reminders showed positive effects on TB cure rates, and no DHT exhibited effects on sputum conversion rates.

Among the primary DHT interventions, VDOT and VOT are the interventions with the potential for improving TB medication adherence. With video-observed treatment, medication adherence can be directly recorded (VDOT) or indirectly (VOT) while a patient takes the medication. Hence, the health care provider can monitor the medication intake process remotely. However, this technology needs adequate smartphone specifications (eg, picture resolution, memory), good connectivity, and user ability. Smartphones with good connectivity are required because each video should be sent in an adequate video resolution and fairly large file size for proper drug monitoring. The benefit of video treatment is that patients’ and health care workers' travel time can be significantly decreased compared to traditional DOT [22]. As an intervention, VDOT or VOT is preferred by patients with TB because it is easier to accept, cheaper, and more effective than is standard DOT [20]. Moreover, the implementation of VDOT or VOT has been associated with treatment completion [20-22] and treatment adherence [24] in patients with TB. Our review found that 2 VDOT studies significantly enhanced treatment completion compared to DOT [20,22], while 2 other studies demonstrated no difference between VDOT and standards of care [21,24]. The challenges concerning its implementation are the availability of (smart) phones and the network-related interruption of audio-video connections during VDOT sessions. However, the widespread use of smartphones will encourage providers to improve network quality. Therefore, VDOT or VOT can be a promising approach to overcoming medication adherence problems [32], especially in countries with high smartphone use [33,34]. The average availability of smartphones in the top-30 high-burden TB countries is 93.98 per 100 people, ranging from 14.9 in North Korea to 185.69 in Montenegro [35]. The data highlighted the potential use of DHT interventions based on smartphone intervention.

Beyond VDOT, SMS reminders were one of the most frequently studied DHT interventions. Phone call reminders are used to remind patients directly to take their medicine. The technology used in this approach is simpler than that of VDOT or VOT because the phone call reminder does not need high device specifications and patient ability; thus, patients can easily use it. One study in Thailand that evaluated 2 models for TB control, single DOT and DOT with phone call reminders, showed that phone call reminders can increase the cure rate [29]. The reminders supported the patients during their treatment because they did not feel alone or socially isolated. Another type of reminder-based intervention is the medication monitor box. This device can also record the history of medicine usage and can be used for adherence monitoring by health care providers at a distance [18]. The medication reminder feature can overcome forgetfulness in patients with TB. A study conducted in 36 districts in China using a medication monitor box with a reminder exhibited a lower missed-dose rate than did a medication monitor box without a reminder [17]. Another RCT conducted in Haiti reported that the medication monitor box without reminders did not effectively reduce the noncompleted rate among patients with TB [31]. The study highlighted that the reminder feature attached in the medication box could affect the completion rate.

Another DHT is the ingestible sensor. The sensor is attached to the medicine and sends a signal to the operator when the medicine is ingested. If the patient is not taking the medication, the system will automatically send a reminder urging them to take medicine immediately. With this ingestible sensor, the potential adverse effects should be considered since the sensor is attached to the patient. An RCT conducted in the USA on patients with advanced-phase TB indicated that ingestible sensors could increase treatment completion with an accuracy of 99.3%. However, rash and pruritus were reported as side effects in up to 10% of the participants [23]. It would be worthwhile to conduct a cost-effectiveness analysis to assess the increased treatment completion at the additional cost of the ingestible sensors and the side effects.

SMS reminders can be considered and combined with other interventions to improve medication adherence in patients with TB. SMS reminders are sent periodically to encourage patients to take their medicine. This feature does not require high smartphone specifications. Indeed, 3 of the 8 RCT studies conducted in 6 countries indicated that the SMS reminder could increase treatment completion [25], treatment adherence [29], and cure rate [29]. The SMS reminder can be applied widely because of its ease of access, low cost, and ready acceptance by patients. Like the medication monitor box, the SMS reminder plays an essential role in improving patients’ medication schedules [25]. However, 5 studies found no benefit compared to the standard of care [9,10,17,26,28]. This implies that careful consideration should be made before implementing this intervention. Implementation under operational research conditions would be ideal as data would become available to evaluate the benefit of the SMS reminder service.

Some studies have indicated that DHT interventions could improve treatment outcomes and patient adherence. However, some studies exhibited no difference compared to the standard of care [9,10,17,21,24,26,28,31]. The variable effects of DHT interventions can be explained using the heterogeneity of the population, comparison, and outcome definitions used across the included studies. For instance, although the same type of intervention and outcome was applied, the use of various comparisons (DOT and standard care without DOT) [10,25] makes it challenging to aggregate results. Notably, the features of the population should be taken into consideration when interpreting the intervention effect on adherence. As nonadherence can be explained using multiple factors (eg, social, economic, and behavioral factors) [4], it is essential to characterize the individual problem of nonadherence when conducting the intervention. Hence, a one-size-fits-all intervention to resolve nonadherence is unlikely to be successful. Therefore, a tailored and targeted intervention for improving medication adherence is essential to resolving nonadherence.

### Implications and Future Directions

Given the contradicting study outcomes, more research and developments regarding the role of DHTs in medication adherence management for patients with TB are needed. These developments should focus on the (technical) drug monitoring aspects and the intervention(s) that can effectively improve medication adherence and treatment outcome. Considering the multiple factors underlying medication nonadherence in patients with TB (eg, social, economic, geographic, facility, and behavioral factors) [4], a tailored and targeted intervention for improving medication adherence is essential. Therefore, a DHT with screening and monitoring for nonadherent patients can be further enhanced by considering the individual problems patients encounter. Subsequently, these data can further support health care providers in effectively delivering personalized interventions for improving medication adherence in patients with TB. This may also require further training of health care providers in effective communication strategies.

Most DHT-based interventions in this study were assessed under trial conditions that may not represent the real-world condition for implementation. Numerous factors may drive the effect of a DHT’s intervention in the real world: population density, facilities, transportation, smartphone network coverage, health care systems, human resources, costs, individual characteristics, and integration of the intervention with the national TB program [36]. This underlines the fact that implementing a particular intervention will have very different meanings depending on the local context. Therefore, understanding the local context is critical for successful DHT intervention implementation in real-world settings. Additionally, governmental policy and proper reimbursement are needed to support and regulate DHTs for successful implementation. Further implementation studies are needed to evaluate the maturity of interventions in the real world to scale up DHT interventions beyond trial settings.

### Strengths and Limitations

This study’s strength is that a comprehensive and up-to-date overview of existing DHTs, ranging from their use, device specifications, and efficacy that can be used to improve the problem of medication adherence and treatment outcome among patients with TB, was provided. However, although all efforts were made to provide a robust analysis, several limitations should be acknowledged: although unpublished RCT studies were covered in the Cochrane Database, potential publication bias may exist in this study due to limited inclusion of gray literature, non–English language studies, and studies indexed in other databases; a meta-analytical analysis could not be conducted due to the heterogeneity across the included studies; as the focus was on RCT studies—known as the gold standard in analyzing the effects of interventions—well-designed pre-and postintervention studies were excluded in this study.

### Conclusions

It was found that DHT can be a promising approach in improving medication adherence and treatment outcomes among patients with TB despite the variable intervention effects that were discovered. Considering individual factors of nonadherence to medication among patients with TB, developing DHT interventions with personalized feedback is required to have a consistent and beneficial effect on medication adherence and treatment outcomes among patients with TB. Further implementation studies are needed to evaluate the maturity and scale-up of the interventions in the real world.

## Appendix

Multimedia Appendix 1 The PRISMA checklist.

Multimedia Appendix 2 The Jadad score table.

## Abbreviations

DHT: digital health technology
DOT: directly observed therapy
EMERGE: ESPACOMP Medication Adherence Reporting Guideline
LTBI: latent tuberculosis infection
M. tb: Mycobacterium tuberculosis
OR: odds ratio
PRISMA: preferred reporting items for systematic review and meta-analysis
RCT: randomized controlled trial
RR: relative risk
TB: tuberculosis
VDOT: video directly observed therapy
VOT: video-observed therapy
WHO: World Health Organization
