# Supplementary material for: Digital Health Technologies to Improve Medication Adherence and Treatment Outcomes in Patients With Tuberculosis: Systematic Review of Randomized Controlled Trials
Source: J Med Internet Res. 2022 Feb 23;24(2):e33062. doi: 10.2196/33062 (PMC8908199; doi:10.2196/33062)
Supplement: Multimedia Appendix 2 [file jmir_v24i2e33062_app2.docx]

| **Appendix 2.** Risk of bias assessment for randomized studies using the JADAD score | | | | | | | |
| --- | --- | --- | --- | --- | --- | --- | --- |
| No | First author and year of publication | Randomization | Description of randomization | Double-blind method | Description of the blinding method | Description of participant withdrawal/dropout | Total score |
| 1 | Story et al., 2019 | 1 | 1 | 0 | 0 | 1 | 3 |
| 2 | Chuck et al., 2016 | 1 | 0 | 0 | 0 | 1 | 2 |
| 3 | Lam et al., 2018 | 1 | 0 | 0 | 0 | 1 | 2 |
| 4 | Chen et al., 2020 | 1 | 0 | 0 | 0 | 1 | 2 |
| 5 | Kunawararak et al., 2011 | 1 | 0 | 0 | 0 | 1 | 2 |
| 6 | Liu et al., 2015 | 1 | 1 | 0 | 0 | 1 | 3 |
| 7 | Moulding & Caymittes, 2002 | 1 | 1 | 0 | 0 | 1 | 3 |
| 8 | Browne et al., 2019 | 1 | 1 | 0 | 0 | 1 | 3 |
| 9 | Fang et al., 2017 | 1 | 1 | 0 | 0 | 1 | 3 |
| 10 | Mohammed et al., 2016 | 1 | 1 | 0 | 0 | 1 | 3 |
| 11 | Belknap at al., 2017 | 1 | 1 | 0 | 0 | 1 | 3 |
| 12 | Bediang et al., 2018 | 1 | 1 | 0 | 0 | 1 | 3 |
| 13 | Johnston et al., 2018 | 1 | 1 | 0 | 0 | 1 | 3 |
| 14 | Iribarren et al., 2013 | 1 | 1 | 0 | 0 | 1 | 3 |
| 15 | Ali & Martin, 2019 | 1 | 0 | 0 | 0 | 1 | 2 |
| 16 | Farooqi et al., 2017 | 1 | 1 | 0 | 0 | 1 | 3 |
| The JADAD questions: (1) Was the study described as randomized?; (2) Was the method used to generate the sequence of randomization described and appropriate?; (3) Was the study described as double-blind?; (4) Was the method of double-blinding described and appropriate?; (5) Was there a description of withdrawals and dropouts? A double-blinding method was either not possible or not applied for the included studies. | | | | | | | |
